# Activation of EP4 alleviates AKI-to-CKD transition through inducing CPT2-mediated lipophagy in renal macrophages

**DOI:** 10.3389/fphar.2022.1030800

**Published:** 2022-11-16

**Authors:** Xu Guan, Yong Liu, Wang Xin, Shaozong Qin, Shuiqin Gong, Tangli Xiao, Daohai Zhang, Yan Li, Jiachuan Xiong, Ke Yang, Ting He, Jinghong Zhao, Yinghui Huang

**Affiliations:** The Key Laboratory for the Prevention and Treatment of Chronic Kidney Disease of Chongqing, Department of Nephrology, Chongqing Clinical Research Center of Kidney and Urology Diseases, Xinqiao Hospital, Army Medical University (Third Military Medical University), Chongqing, China

**Keywords:** AKI-to-CKD transition, EP4, CPT2, lipophagy, macrophages

## Abstract

Acute kidney injury (AKI) is a common clinical syndrome with complex pathogenesis, characterized by a rapid decline in kidney function in the short term. Worse still, the incomplete recovery from AKI increases the risk of progression to chronic kidney disease (CKD). However, the pathogenesis and underlying mechanism remain largely unknown. Macrophages play an important role during kidney injury and tissue repair, but its role in AKI-to-CKD transition remains elusive. Herein, single nucleus RNA sequencing (snRNA-Seq) and flow cytometry validations showed that E-type prostaglandin receptor 4 (EP4) was selectively activated in renal macrophages, rather than proximal tubules, in ischemia-reperfusion injury (IRI)-induced AKI-to-CKD transition mouse model. EP4 inhibition aggravated AKI-to-CKD transition, while EP4 activation impeded the progression of AKI to CKD though regulating macrophage polarization. Mechanistically, network pharmacological analysis and subsequent experimental verifications revealed that the activated EP4 inhibited macrophage polarization through inducing Carnitine palmitoyltransferase 2 (CPT2)-mediated lipophagy in macrophages. Further, CPT2 inhibition abrogated the protective effect of EP4 on AKI-to-CKD transition. Taken together, our findings demonstrate that EP4-CPT2 signaling-mediated lipophagy in macrophages plays a pivotal role in the transition of AKI to CKD and targeting EP4-CPT2 axis could serve as a promising therapeutic approach for retarding AKI and its progression to CKD.

## Introduction

Acute kidney injury (AKI), defined as a rapid decline in renal function, is a common clinical syndrome with high mortality, morbidity and complex pathogenesis in hospitalized patients (Valino-Rivas et al., 2022; Yao et al., 2022). Moreover, increasing evidence has demonstrated that incomplete recovery from AKI boostes the risk of progression to chronic kidney disease (CKD) (Carney, 2021; Fu et al., 2022). As reported, approximately 30%–70% of AKI patients progress to CKD and end-stage renal disease (ESRD) (Guzzi et al., 2019). Due to the complex etiology of AKI, the pathogenesis and molecular mechanism remain unclear, and therapeutic interventions are in urgent need.

Numerous studies indicate that macrophages are a major contributor to the inflammatory response of AKI (Huen and Cantley, 2017; Martin-Sanchez et al., 2022). Macrophage-induced inflammatory and fibrotic responses are also key drivers of renal fibrosis, which will enhance the progression of CKD (Chen et al., 2022; Zhou et al., 2022). Responding to tissue damage, macrophages are activated in response to particular signals from the injury microenvironment (Okubo et al., 2018). Previous studies have demonstrated that the activation and functional status of macrophages depend on the phase of tissue damage and repair, reflecting a dynamic and diverse macrophage phenotype. In general, macrophages can differentiate into a pro-inflammatory phenotype in response to the early kidney injury, while during the repair phase, macrophages can differentiate into an anti-inflammatory phenotype, which counteracts the effects of abnormal inflammation and supports tubule regeneration (Zheng et al., 2021b; Bhatia et al., 2022). Thus, macrophages are a potential therapeutic target for AKI. However, the molecular mechanism of macrophages in the progression of AKI to CKD remains unclear.

Previous studies have shown that prostaglandin E2 (PGE2) plays an important role in AKI and CKD, which signals primarily through four G protein-coupled receptors, including E-type prostaglandin receptor (EP) one to EP4 (Crittenden et al., 2021; Minhas et al., 2021). EP4 is the major PGE2 receptor in macrophages, and activation of EP4 in macrophages inhibits the release of pro-inflammatory cytokines and chemokines (Pan et al., 2022a). Recent studies have reported that myeloid cyclooxygenase (COX)-2-derived PGE2 modulates renal myeloid cell polarization through EP4 receptor (Pan et al., 2022b), hinting that EP4 activation exerts reno-protective effect on AKI mice. However, the role and underlying mechanism of EP4-regulated macrophage polarization is not fully understood.

Lipophagy, also known as autophagic degradation of lipids, is a key mechanism regulating lipid metabolism in a variety of cells (Han et al., 2021; Shroff and Nazarko, 2022). Lipophagy, as a specialized type of autophagy, selectively degrades intracellular cholesterol and triglycerides (TGs) stored in lipid droplets (LDs) by lysosomal acid lipase (Cui et al., 2021). Recent evidence confirms that AKI is closely related to significant abnormal metabolism, especially lipid metabolism abnormalities (Bugarski et al., 2021; Xiong et al., 2021; Kim et al., 2022). However, current research on AKI mainly focuses on lipid metabolism in proximal tubules cells, whereas lipid metabolism in macrophages in the progression of AKI-to-CKD transition has not been thoroughly investigated.

In the present study, we found that EP4 was selectively activated in renal macrophages but not in proximal tubules after ischemia-reperfusion injury (IRI). Aberrant activation of EP4 in renal macrophage was closely involved in AKI and progression of CKD. Promoting EP4 activation impeded AKI to CKD progression though regulation of macrophage polarization. Mechanistically, EP4 in macrophages regulated macrophage polarization through activation of Carnitine palmitoyltransferase 2 (CPT2)-mediated lipophagy, ameliorated IRI, mitigated renal fibrosis, and prevented AKI-to-CKD transition. Taken together, these findings identify EP4-CPT2 signaling could serve as a promising therapeutic target for retarding AKI and its progression to CKD.

## Materials and methods

### Single nucleus RNA sequencing analysis

We downloaded kidney snRNA-seq dataset of the sham and IRI mice from the NCBI GEO database (Accession number GSE139107) (Kirita et al., 2020). The quality control of the snRNA-seq dataset as follows: gene number between 500 and 4,000, UMI count >1,000 and the mitochondrial gene percentage <0.1. The doublet cells were found using the Doublet Finder (McGinnis et al., 2019) and doublets >0.5 were excluded. Harmoniously integrate the matrices of all samples (Korsunsky et al., 2019) to eliminate batch effects between different samples. In terms of parameter setting, Seurat3 software was used to set parameters for the first 30 dimensions of canonical correlation analysis and principal component analysis (PCA) (Stuart et al., 2019).

### Animal study

The AKI-to-CKD mouse model refers to previous studies (Xiong et al., 2022). Briefly, 8–12 weeks male mice were anesthetized, followed by an abdominal incision, and the renal pedicles of both kidneys were clamped for 32 min to induce IRI. Mice were to maintain a constant temperature (37°C) during the procedure of ischemia. The sham group only exposed the kidneys with no additional closure. Mice were sacrificed at 1, 3, 7, and 14 days after IRI, and blood and kidney tissue samples were collected for subsequent analysis. Administration and Dosage of agonists and inhibitors according to previous studies (Vella et al., 2015; Watanabe et al., 2015; Lannoy et al., 2020; Hu et al., 2022). ONO-AE3-208 (10 mg/kg/day, intraperitoneally), CAY10580 (3 mg/kg/day, oral administration), 3-MA (15 mg/kg/day, intraperitoneally), Perhexiline maleate (3 mg/kg/day, oral administration). The animal study was approved by the Animal Ethics Review Board of the Army Medical University.

### Flow cytometric analysis

Mice were sacrificed to prepare single-cell suspensions of kidney cells after IRI. After removing red blood cells, cells were wash with PBS. Zombie was used to distinguish living cells. After blocking with CD16/32, cell suspensions are incubated with primary antibodies. Incubate for 15 min at a concentration of 1:100 at 4°C from light. Flow cytometric analysis were analyzed using a ID7000 (Sony, Japan) flow cytometer. Primary macrophage isolation was labelled and separated using the same antibodies. Data analysis was conducted by Flow Jo software (Treestar Inc., United States). The antibody used in this study is listed in Supplementary Table S1.

### Scr and BUN measurements

Scr and BUN were detected using a commercial kit (NJJCBIO, China) according to previously reportt following manufacturer’s instructions (Xiong et al., 2022).

### Cell culture

Human monocytes THP-1 cells and HK-2 cells were obtained from American type culture specimens (ATCC, Manassas, VA, United States) and cultured in RPMI-1640 cell or DMEM/F12 culture medium Supplement with 10% FBS at 37°C in a humidified atmosphere with 5% CO2. To further determine the effects of CAY10580 or ONO-AE3-208 on THP-1 cells, THP-1 cells were induced to macrophages in the presence of Phorbol 12-myristate 13-acetate PMA (100 ng/ml) for 24 h, subsequently exposed to CAY10580, ONO-AE3-208, 3-MA or Perhexiline maleate for 24 h. M1 or M2 macrophages were polarized by stimulating with 10 ng/ml lipopolysaccharide (Sigma-Aldrich) and 20 ng/ml IFN-γ (peprotech) or 20 ng/ml IL-4 (peprotech) after incubated with PMA for 24 h.

### qRT-PCR

Total RNA was extracted from THP-1 cells and mouse kidney tissue by using RNA extraction kit (Beyotime, Shanghai, China). Reverse transcription and mRNA expression detection was executed using reverse transcription kit and SYBR Green PCR Master mix (MCE, United States). The primer sequences are listed in Supplementary Table S2.

### Target gene prediction

The potential target genes of EP4 agonist and inhibitor were predicted by SwissTargetPrediction (http://www.swisstargetprediction.ch). AKI-to-CKD related genes were predicted by Genecards (https://www.genecards.org).

### Western blot

Proteins of cultured THP-1 cells and kidney tissue samples were isolated and separated by SDS-PAGE. After transferred to polyvinylidene fluoride (PVDF) membranes (Millipore, Billerica, MA, United States), the samples were blocked with Quick blocking solution (Beyotime, China). Then incubate with primary antibody overnight at 4°C and the secondary antibodies 1 h for 37°C. and the signals were detected by enhanced chemiluminescence. The antibody is listed in Supplementary Table S1.

### Histology and immunohistochemical staining

Paraffin-embedded sections of mouse kidneys were prepared by using conventional methods. Sections were stained with H&E and Masson’s trichrome to detect kidney injury and fibrosis. Quantitative analysis of fibrotic areas was performed using ImageJ software (Bethesda, MD, United States). Immunostaining was performed as described previously (Liu et al., 2019). Briefly, after dewaxing, hydration, endogenous enzyme, and biotin removal and antigen repair, the sections were incubated with primary antibodies at 4°C overnight, followed by incubation with secondary antibody and 3,3′-diaminobenzidine (DAB) for color development. The antibody is listed in Supplementary Table S1.

### Transmission electron microscope

Transmission electron microscope (TEM) (JEM-1400PLUS, Japan) was used for autophagosome-encapsulated lipid droplets observation. Briefly, cells were fixed with 3% glutaraldehyde followed by 2% osmic acid. Gradient dehydration, acetone-embedded cells.

### Cell transfection

Cell transfection refers to our previous transfection method (Liu et al., 2019). The overexpression plasmid of CPT2 was purchased from Youbio (Hunan, China). mCherry-GFP-LC3 adenovirus transfection refers to previous studies (Huang et al., 2020).

### Oil Red O staining

THP-1 cells were treated with PMA (100 ng/ml) for 24 h and then fixed with 4% paraformaldehyde for 15 min after treated with CAY10580 or ONO-AE3-208, following stained with 60% isopropanol Oil Red O solution for 10 min. After washed with distilled water, counterstained with Mayer’s hematoxylin, then observed with a light microscope and photographed. HK-2 cells were treated with CAY10580, ONO-AE3-208 for 24 h and then following above procedure.

### Statistical analysis

All data were presented as mean ± SD. Unpaired *t* test or one-way analysis of variance (ANOVA) with Tukey’s test was used for statistical analysis. Statistical significance was defined as *p* < 0.05.

## Results

### EP4 was selectively activated in renal macrophages after ischemia-reperfusion injury

**FIGURE 1:**
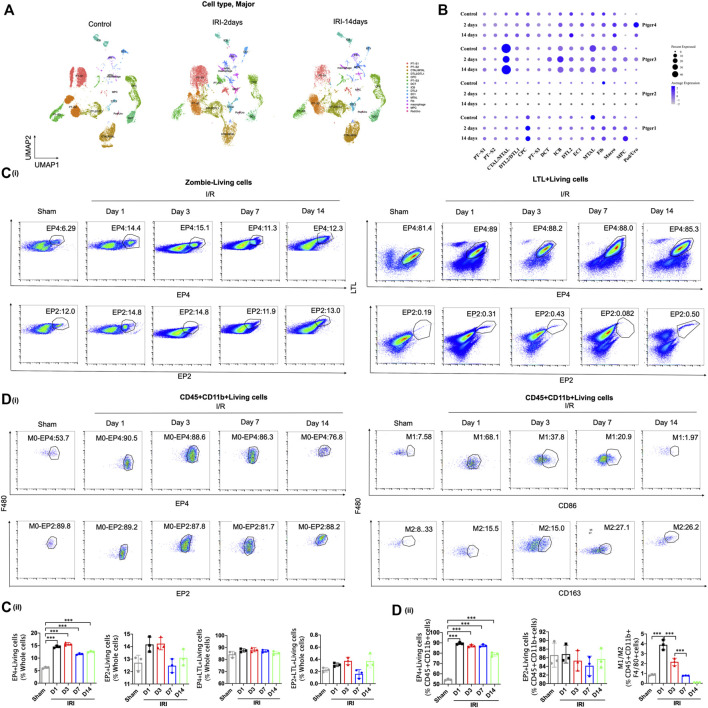
EP4 is selectively activated in renal macrophages after ischemia-reperfusion injury. **(A–B)** Cell Types and Clusters Identified by snRNA-seq in Kidney Tissues of IRI mice. t-Distributed stochastic neighbor embedding (t-SNE) plot showing major cell clusters or type. Cell clusters were identified by kidney cell lineage-specific marker expression. PT-S1, the S1 segment of proximal tubule; PT-S2, the S2 segment of proximal tubule; CTAL, thick ascending limb of loop of Henle in cortex; DTL, descending limb of loop of Henle; CPC, principle cells of collecting duct in cortex; PT-S3, S3 segment of proximal tubule; DCT, distal convoluted tubule; ICB, type B intercalated cells of collecting duct; EC, endothelial cells; MTAL, thick ascending limb of loop of Henle in medulla; Fib, fibroblasts; Macro, macrophages; Pod, podocytes; MPC, principle cells of collecting duct in medulla; Uro, urothelium. **(C)** Flow cytometric analysis of the percentage of EP4 and EP2 living cells (Zombie^−^) in whole kidney cells of AKI mice at 1, 3, 7, and 14 days after ischemia-reperfusion injury. Flow cytometric analysis of the percentage of EP4 and EP2 LTL^+^living cells (Zombie^−^LTL^+^) in whole kidney cells of AKI mice. **(D)** Flow cytometric analysis of the percentage of EP4 and EP2 in renal macrophages (Zombie^−^CD45^+^CD11b^+^F4/80^+^cells) in kidney of AKI mice. Flow cytometric analysis of the percentage of M1 (CD86^+^) and M2 (CD163^+^) in renal macrophages (Zombie^−^CD45^+^CD11b^+^F4/80^+^cells) in kidney of AKI mice. The percentage of cells in kidney from IRI mice by flow cytometry. *n* = 3 mice per group, Data are means ± s.d. ***p < 0.001, **p < 0.01, *p < 0.05.

To explore the potential role of EP receptors in the procedure of AKI-to-CKD transition, we first screened the expressions of the four G-protein-coupled receptors EP receptors in mouse kidney tissues at 2 and 14 days after IRI though single nucleus RNA sequencing (snRNA-Seq) acquired from GEO datasets (GSE139107) (Figure 1A). Although both EP3 and EP4 were highly expressed in the kidney (Figure 1B), previous studies have confirmed that EP3 was not significantly altered in the kidney of AKI mice (Abouelkheir et al., 2021). Therefore, we focused on EP4 in subsequent experiments. The snRNA-Seq data showed that EP4 was selectively and gradually upregulated in renal macrophages in IRI-induced AKI-to-CKD mouse model, whereas the expressions of EP1, EP2 and EP3 had no consistent change (Figure 1B), hinting that EP4 in macrophages might play a significant role in the progression of AKI to CKD. To validate the above snRNA-Seq results, flow cytometry was then performed, which further confirmed that, the expression of EP4 was significantly increased in the kidney after IRI, whereas there were no obvious changes in EP2 expression between the sham and IRI group (Figure 1B, Supplementary Figures S1A–C). Moreover, there were no obvious changes between sham and IRI group in the expressions of EP4 and EP2 in proximal tubular cells labeled by a proximal tubular marker, lotus tetragonolobus lectin (LTL) (Figure 1C), which was consistent with the snRNA-Seq data (Figure 1B). Furthermore, EP4 expression in macrophages, marked by CD45, CD11b and F4/80, was significantly increased after IRI, whereas the expression of EP2 in CD45^+^CD11b^+^F4/80^+^ macrophages had no significant change (Figure 1D). Notably, the increased EP4 was predominantly co-localized with CD68^+^ or CD163^+^ cells, the hallmarks of M1/M2 macrophages in the kidney of IRI mice, indicating that EP4 might regulate the polarization of macrophages. Taken together, EP4 is selectively activated in macrophages of kidney tissues from AKI mice, and EP4 activation is engaged in AKI-to-CKD transition.

### EP4 inhibition aggravates AKI-to-CKD transition

**FIGURE 2:**
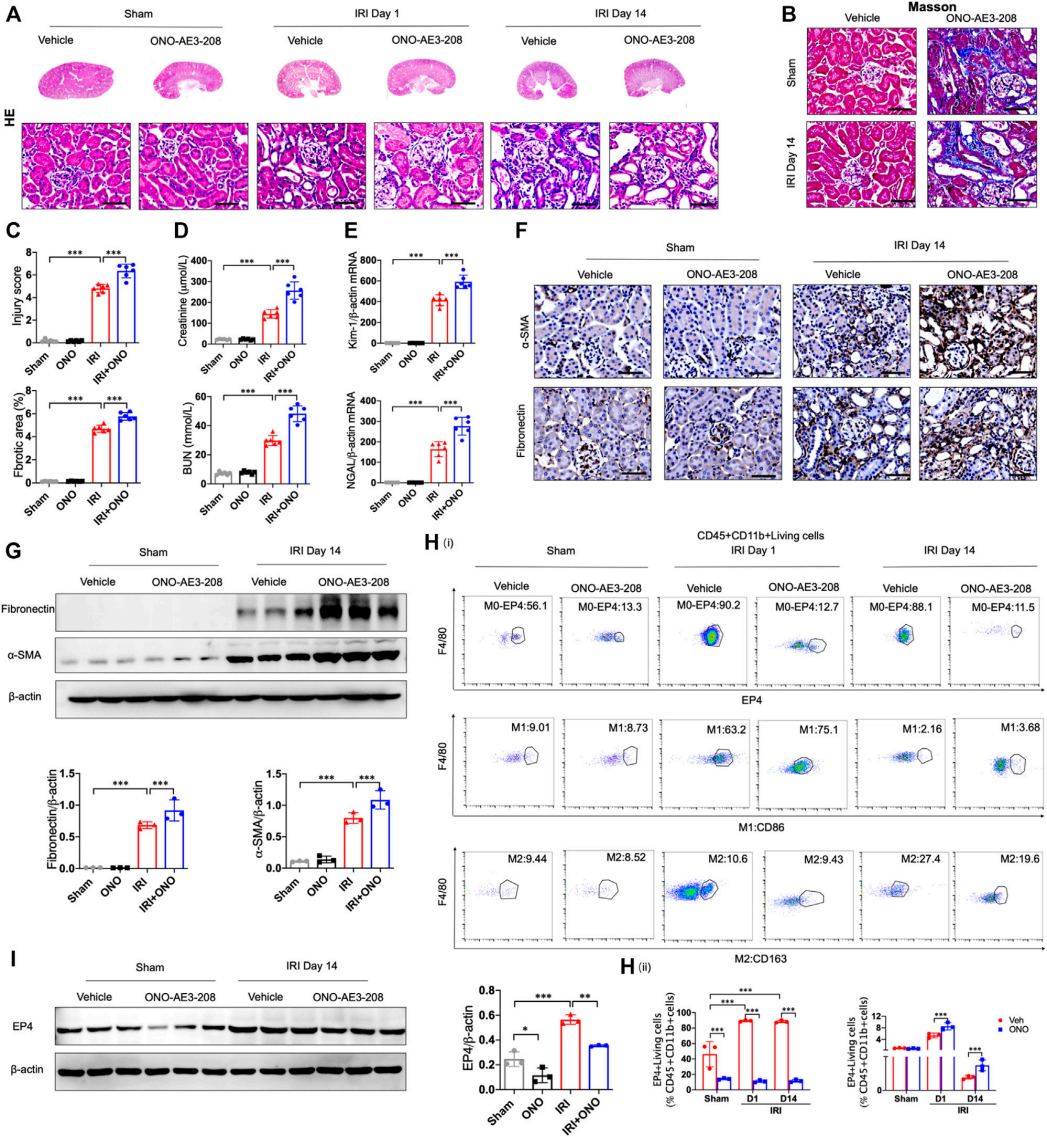
EP4 inhibition aggravates AKI-to-CKD transition. **(A,B)** Representative micrographs of HE and Masson staining of kidney sections from sham and IRI-induced AKI mice injected with control or EP4 inhibitor ONO-AE3-208. **(C)** Scoring of injury score and fibrotic area according to HE and Masson staining of kidney sections from sham and IRI mice. **(D)** Serum creatinine and blood urea nitrogen levels of sham and IRI mice. **(E)** The mRNA levels of Kim-1 and Ngal in the kidney of sham and IRI mice analyzed by qPCR. **(F)** Representative Immunostaining of fibronectin and α-SMA in kidney sections from sham and IRI mice. **(G)** Western blot analysis for fibronectin and α-SMA protein levels in kidney sections from sham and IRI mice. **(H)** Flow cytometric analysis of the percentage of EP4, M1 (CD86^+^) and M2 (CD163^+^) in renal macrophages (Zombie^−^CD45^+^CD11b^+^F4/80^+^cells) in kidney of sham and IRI mice. **(I)** Western blot analysis for EP4 protein levels in kidney sections from sham and IRI mice treated with ONO-AE3-208. Scale bars, 50 μm. *n* = 6 mice per group. Data are means ± s.d. ***p < 0.001, **p < 0.01, *p < 0.05.

To determine the responsibility of EP4 in the process of AKI to CKD, we sought to inhibit EP4 in the kidney after AKI by using an EP4 specific inhibitor, ONO-AE3-208. Compared with the sham group, HE and Masson staining showed the kidney injury, inflammatory infiltration, and subsequent interstitial fibrosis in kidney tissues from IRI-induced AKI-to-CKD mouse models, which were significantly exacerbated by EP4 inhibitor (Figures 2A–C). Consistently, the levels of Scr, BUN and the expressions of Kidney injury molecule-1 (Kim-1) and Neutrophil gelatinase-associated lipocalin (Ngal), AKI markers, were markedly elevated in AKI mice, which were significantly aggravated after EP4 inhibitor treatment (Figures 2D,E). Immunohistochemical and western blot analyses showed that the expressions of fibrosis markers, fibronectin and α-SMA, were significantly increased at 14 days after AKI, while all these changes were aggravated in EP4 inhibitor-treated IRI mice (Figures 2F,G). We also confirmed that EP4 inhibitor indeed restricted the expression of EP4 in renal tissues after AKI injury by western blot and flow cytometry analysis (Figures 2H,I). To explore the potential mechanism of EP4 activation in AKI-to-CKD transition, flow cytometry was applied to determine the influence of EP4 activation on macrophage polarization. As showed in Figure 2H, EP4 inhibitor significantly inhibited the activation of EP4 in macrophages, suppressed the pro-inflammatory M1 macrophages, and increased the anti-inflammatory M2 macrophages in AKI mice, compared with vehicle-treated group. Collectively, these results suggest that inhibition of EP4 accelerate AKI-to-CKD transition *via* enhancing renal macrophage polarization.

### Sustained activation of EP4 restrains the progression of AKI to CKD

**FIGURE 3:**
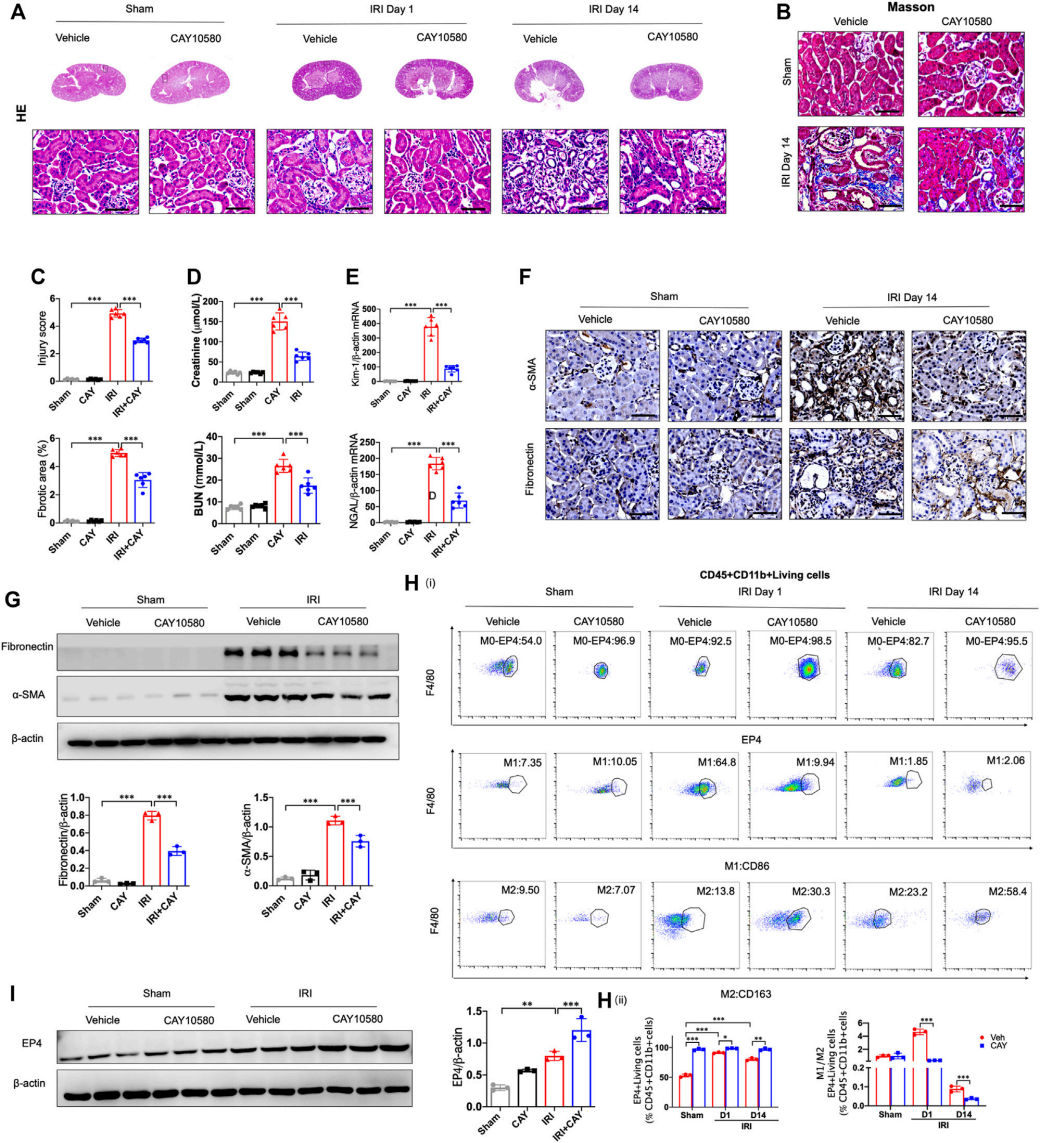
Sustained activation of EP4 restrains the progression of AKI to CKD. **(A,B)** Representative micrographs of HE and Masson staining of kidney sections from sham and IRI-induced AKI mice injected with control or CAY10580, a EP4 agonist. **(C)** Scoring of injury score and fibrotic area according to HE and Masson staining of kidney sections from sham and IRI mice. **(D)** Serum creatinine and blood urea nitrogen levels of sham and IRI mice. **(E**) The mRNA levels of Kim-1 and Ngal in the kidney of sham and IRI mice analyzed by qPCR. **(F)** Representative Immunostaining of fibronectin and α-SMA in kidney sections from sham and IRI mice. **(G)** Western blot analysis for fibronectin and α-SMA protein levels in kidney sections from sham and IRI mice. **(H)** Flow cytometric analysis of the percentage of EP4, M1 (CD86^+^) and M2 (CD163^+^) in renal macrophages (Zombie^−^CD45^+^CD11b^+^F4/80^+^cells) in kidney of sham and IRI mice. (*n* = 3 mice per group). **(I)** Western blot analysis for EP4 protein levels in kidney sections from sham and IRI mice treated with CAY10580. Scale bars, 50 μm, *n* = 6 mice per group, Data are means ± s.d. ***p < 0.001, **p < 0.01, *p < 0.05.

To further identify the influence of sustained activation of EP4 on the progression of AKI to CKD, EP4 agonist was employed in the subsequent studies. As shown in Figures 3A–E, the swelling and vacuolization of renal tubules as well as interstitial fibrosis were dramatically decreased in the EP4 agonist (CAY10580)-treated group, accompanied by a reduction of the levels of Scr, BUN, Kim-1 and Ngal, compared with the vehicle group. Simultaneously, the expressions of fibronectin and α-SMA were dramatically decreased in AKI mice at 14 days after EP4 agonist treatment (Figures 3F,G). Flow cytometry and western blot analyses also confirmed the enhanced expression of EP4 in the kidneys of IRI mice treated with EP4 agonist, accompanied by an inhibition of M1 macrophages, and the increased M2 macrophages in AKI mice (Figures 3H,I). Given the important role of macrophages in AKI-to-CKD transition, the above results clearly indicate that EP4 agonist possess a renal protective role on kidney function and renal fibrosis after IRI injury, suggesting that sustained activation of EP4 impedes AKI-to-CKD progression though regulating macrophage polarization.

### EP4 inhibits macrophage polarization *via* inducing lipophagy

**FIGURE 4:**
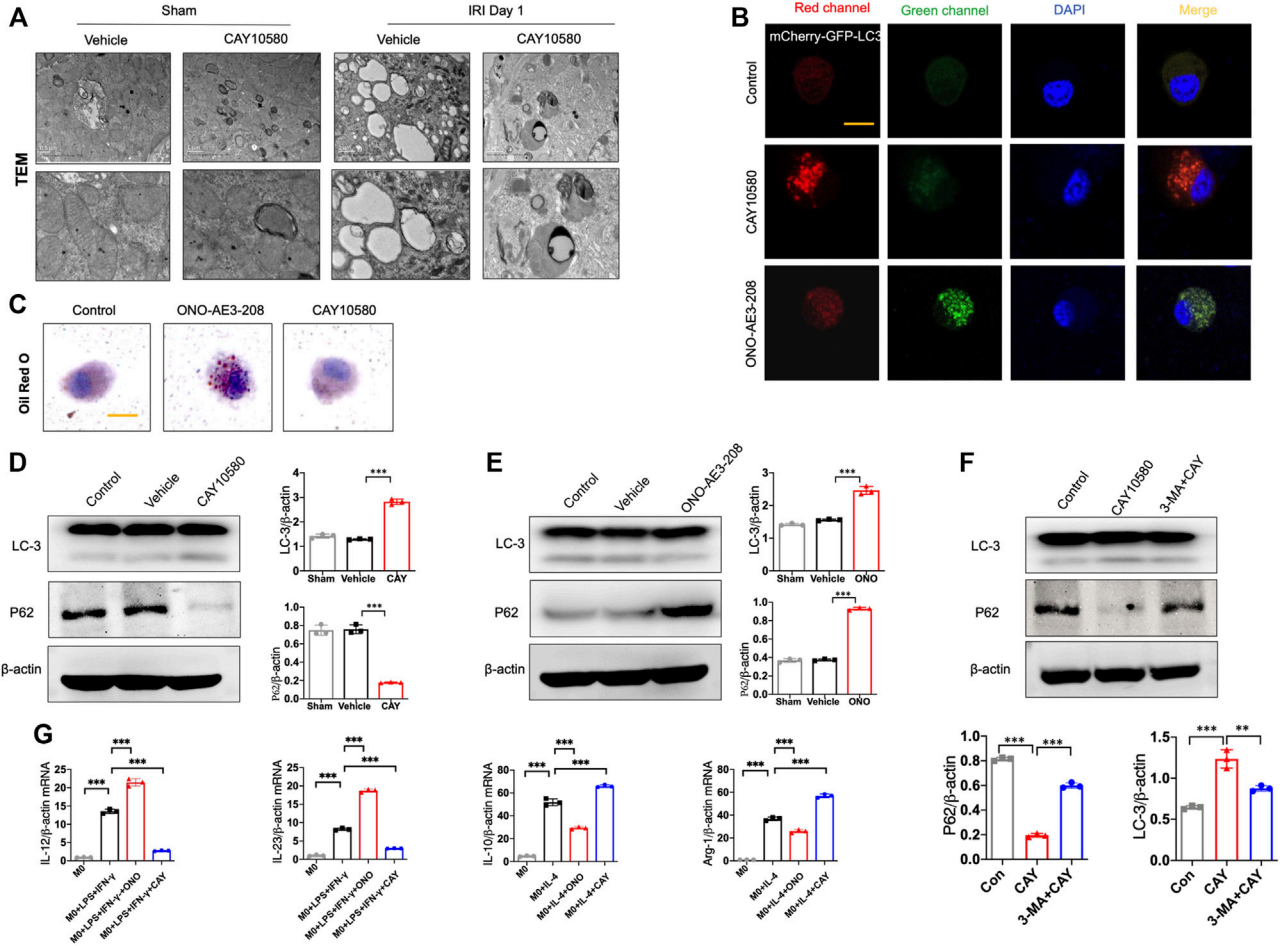
EP4 inhibits macrophage polarization via inducing lipophagy. **(A)** Representative images of transmission electron microscope (TEM) observation of autophagosome-encapsulated lipid droplets of the kidney from sham and IRI mice. **(B)** Representative Immunostaining of mCherry-GFP-LC3 in THP-1 cells treated with CAY10580 or ONO-AE3-208. **(C)** Representative micrographs of Oil red O (ORO) staining in THP-1 cells pre-incubated with PMA for 24 h and subsequently treated with treated with CAY10580 or ONO-AE3-208. **(D,E)** Western blot analysis for LC-3 and P62 protein levels in THP-1 cells pre-incubated with PMA for 24 h and subsequently treated with CAY10580 or ONO-AE3-208. **(F)** Western blot analysis for LC-3 and P62 protein levels in THP-1 cells pre-incubated with PMA for 24 h and subsequently treated with CAY10580 or 3-MA. **(G)** qPCR analysis of the mRNA levels of IL-12, IL-23, IL-10 and Arg-1 in THP-1 cells after incubation with PMA following treated with LPS and IFN-γ, or IL-4, respectively. Data are means ± s.d. *n* = 6 mice per group, ***p < 0.001, **p < 0.01.

Previous studies indicated that EP4 was involved in autophagy and mitophagy in other disease models (Ding et al., 2019; Cao et al., 2022). To examine whether EP4 regulate autophagy in macrophages during the progression of AKI to CKD, we assessed the ultrastructural changes in macrophages of kidney after IRI. Interestingly, we TEM observations showed an increase in autophagosome-encapsulated lipid droplets, accompanied with an alleviation of tissues damage in EP4 agonist-treated AKI mice, suggesting that lipophagy might be involved in EP4-mediated effects (Figure 4A). To determine whether EP4 can regulate lipophagy, THP-1 cells, a widely used human monocyte which could differentiate into macrophage-like cells, were used to study the underlying mechanism *in vitro*. Accordingly, red spots were yielded in THP-1 cells transfected with mCherry-GFP-LC3 adenovirus followed by treatment with EP4 agonist, while EP4 inhibitor led to yellow spots (Figure 4B). The accumulation of lipid droplets was induced by EP4 inhibitor, while inhibited by EP4 agonist (Figure 4C). Meanwhile, we also examined lipid accumulation and the expression of Perilipin-2 (PLIN2) in HK-2 cells by Oil Red O Staining and western blot analysis, respectively. EP4 inhibitor led to a slight lipid deposition and an increasing expression of PLIN2 in HK-2 cells, whereas EP4 agonist treatment has no significant influence on HK-2 cells (Supplementary Figure S4). Further, western blot analysis also confirmed that EP4 agonist upregulated LC3-II expression and downregulated the autophagy substrate P62 (also known as sequestosome 1, SQSTM1) expression in THP-1 cells (Figures 4D,E). Conversely, EP4 inhibitor downregulated the expression of LC3-II, upregulated P62 protein level (Figure 4F), indicating that EP4 could induce lipophagy in macrophages. Meanwhile, qRT-PCR was used to detect the expression changes of M1 (IL-12 and IL-23) and M2 (IL-10 and Arg-1) related genes. The expressions of M1 and M2-related genes were significantly increased after THP-1-induced M0 macrophages following treated with LPS and IFN-γ, or IL-4, respectively. EP4 inhibitors significantly increased the expression of M1 related genes and decreased the expression of M2-related genes. Conversely, EP4 agonists significantly down-regulated the expression of M1-related genes and up-regulated the expression of M2-related genes (Figure 4G). These results confirmed that EP4 also regulated the polarization of macrophage-like cells *in vitro*.

**FIGURE 5:**
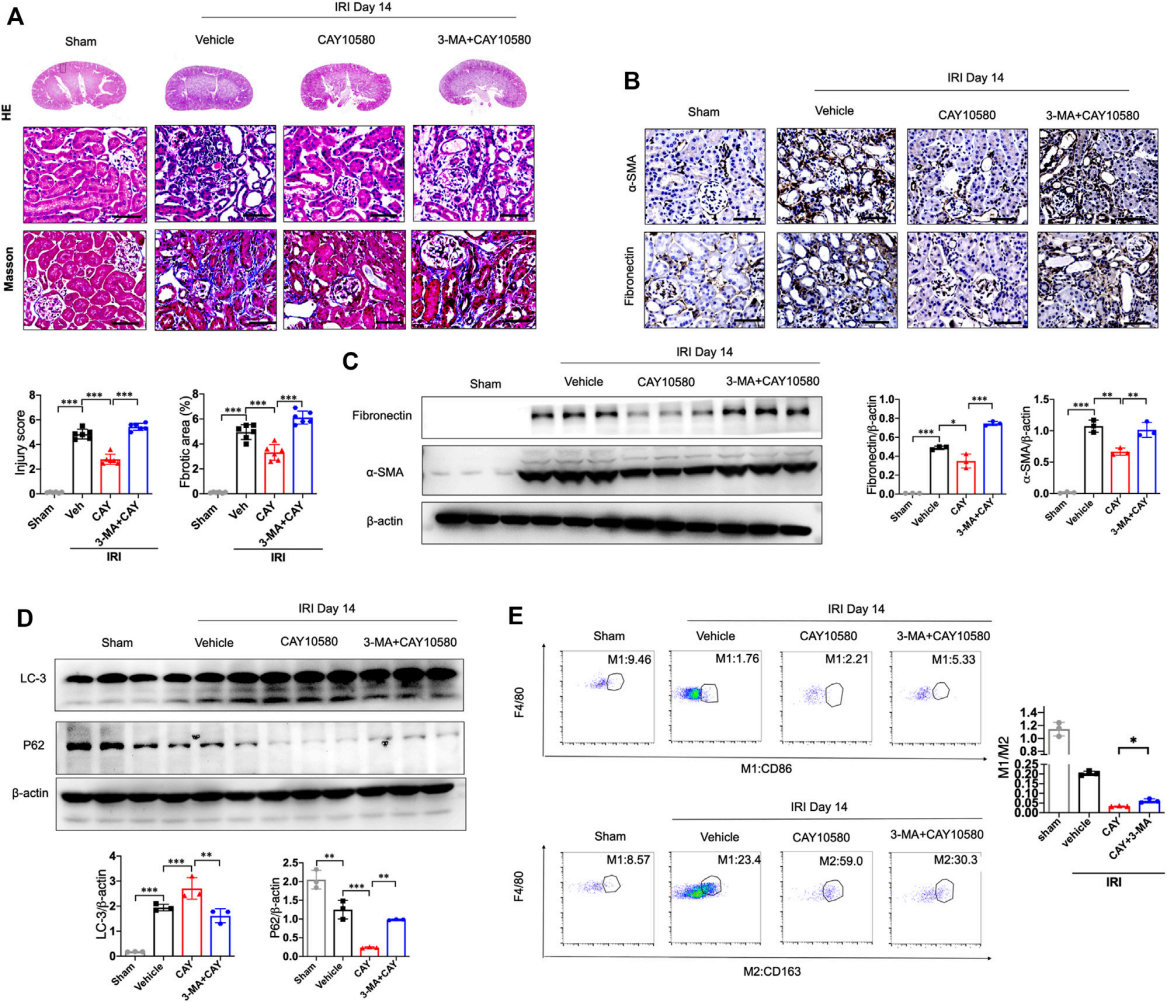
EP4 attenuates AKI-to-CKD transition through inducing lipophagy. **(A)** Representative micrograph of HE and Masson staining of kidney sections from sham and IRI mice treated with CAY10580 or 3-MA. **(B)** Representative Immunostaining of fibronectin and α-SMA in kidney sections from sham and IRI mice treated with CAY10580 or 3-MA. **(C)** Western blot analysis for fibronectin and α-SMA protein levels in kidney sections from sham and IRI mice treated with CAY10580 or 3-MA. **(D)** Western blot analysis for LC-3 and P62 protein levels in kidney sections from sham and IRI mice treated with CAY10580 or 3-MA. **(E)** Flow cytometric analysis of the percentage of M1 (CD86^+^) and M2 (CD163^+^) in renal macrophages (Zombie^−^CD45^+^CD11b^+^F4/80^+^cells) in kidney of sham and IRI mice. (*n* = 3 mice per group). *n* = 6 mice per group, Scale bars, 50 μm, Data are means ± s.d. *n* = 6 mice per group, ***p < 0.001, **p < 0.01.

Furthermore, 3-MA, a classic autophagy inhibitor, could significantly reverse EP4 agonist-induced autophagy, and abrogate the ameliorating effects of EP4 agonist on the progression of AKI to CKD *in vivo*, as evidenced by alleviated kidney injury, reduced fibrotic area, downregulated expressions of fibrosis markers (fibronectin and α-SMA) and increased the pro-inflammatory M1 macrophages, and inhibited the anti-inflammatory M2 macrophages in IRI mice, compared with CAY10580-treated group though inducing lipophagy in macrophages (Figures 5A–E). Together, these findings collectively reveal that EP4 attenuates AKI-to-CKD transition through inducing lipophagy.

### EP4 induces lipophagy through upregulating CPT2 expression in macrophages

**FIGURE 6:**
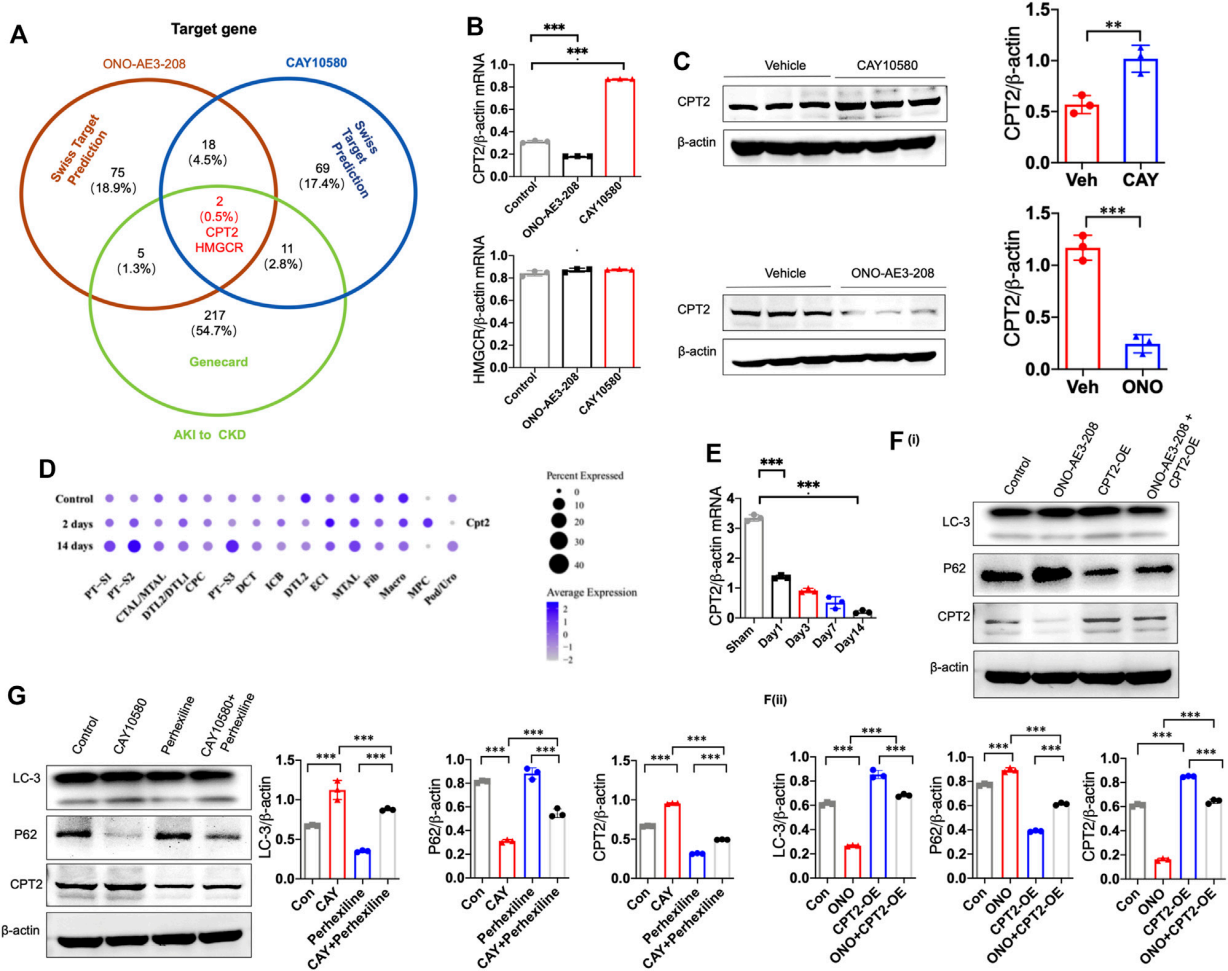
EP4 induces lipophagy through upregulating CPT2 expression in macrophages. **(A)** Genecard and SwissTargetPrediction (network pharmacology analysis) were employed to predict the target genes of EP4. **(B)** qRT-PCR analysis of CPT2 and HMGCR expression in THP-1 cells pre-incubated with PMA for 24 h and subsequently treated with ONO-AE3-208 or CAY10580 for 24 h. **(C)** Western blot analysis of CPT2 expression in THP-1 pre-incubated with PMA for 24 h and subsequently treated with ONO-AE3-208 or CAY10580 for 24 h. **(D)** Dot plot displaying gene expression patterns of cluster-enriched markers in the kidney from sham and IRI mice at 2 days and 14 days after surgery. **(E)** qRT-PCR analysis of CPT2 expression in macrophages separated from the kidney of sham and IRI mice by Flow sorting. **(F,G)** Western blot analysis of CPT2 expression in THP-1 cells pre-incubated with PMA for 24 h subsequently treated with CPT2 specific inhibitor (Perhexiline) or overexpression (OE) plasmids in combined with EP4 agonist or inhibitor, respectively. Scale bars, 50 μm, Data are means ± s.d. ***p < 0.001, **p < 0.01.

To investigate the underlying mechanism by which EP4 regulates lipophagy, we conducted bioinformatic analysis, and predicted two common potential EP4 target genes (CPT2 and HMGCR) based on two different databases, including SwissTarget and Genecards (Figure 6A). Subsequent qPCR screening and western blot analysis demonstrated that CPT2 was upregulated by CAY10580 and downregulated by ONO-AE3-208 (Figures 6B,C), while HMGCR expression was not significantly influenced (Figure 6B). Synchronously, snRNA-Seq data also revealed the reduced expression of CPT2 in renal macrophages of mice with IRI (Figure 6D). Simultaneously, we also analyzed the expression of CPT2 in the macrophages separated from the kidney tissues of IRI mice, which also demonstrated decreased expression of CPT2 (Figure 6E). These data hint that EP4 induces CPT2 expression at both transcriptional level and translational level in macrophages.

To further verify the above results, THP-1 cells were transfected with CPT2 overexpression (OE) plasmids and then treated with vehicle or EP4 agonist or inhibitor, in combined with or without CPT2 inhibitor, respectively. Our results showed that overexpression of CPT2 restored EP4 inhibitor-repressed autophagy, while inhibition of CPT2 markedly suppressed EP4 agonist-induced autophagy in THP-1 cells (Figure 6F).

To further explore whether CPT2 mediates EP4-induced autophagy, we treated THP-1 cells with CPT2 specific inhibitor (Perhexiline) or overexpression (OE) plasmids, respectively. As expected, CPT2 overexpression restored EP4 inhibitor-repressed autophagy (Figure 6F), while CPT2 inhibitor significantly abrogated EP4 agonist-induced autophagy in THP-1 cells and renal tissues from mice with IRI (Figure 6G, Supplementary Figure S2). Taken together, these findings clearly suggest that EP4 enhances lipophagy *via* inducing CPT2 expression in macrophages.

### CPT2 inhibition abrogates the protective effect of EP4 on AKI-to-CKD transition

**FIGURE 7:**
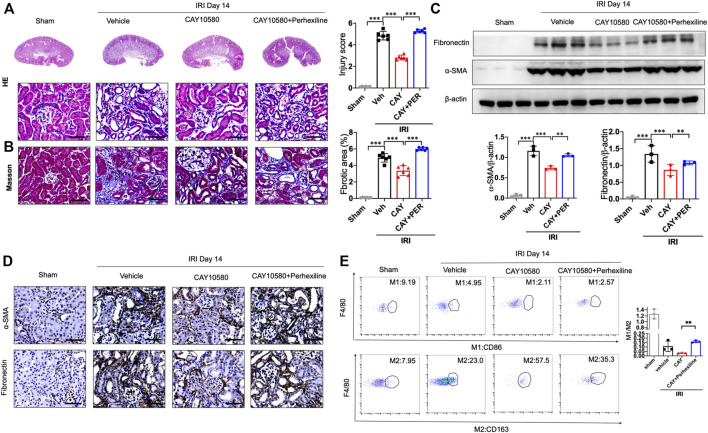
CPT2 inhibition abrogates the protective effect of EP4 activation on AKI-to-CKD. **(A,B)** Representative micrographs of HE and Masson staining of kidney sections from sham and IRI mice treated with ONO-AE3-208 or Perhexiline (maleate). **(C)** Western blot analysis for fibronectin and α-SMA protein levels in kidney sections from sham and IRI mice treated with ONO-AE3-208 or Perhexiline (maleate). **(D)** Representative Immunostaining of fibronectin and α-SMA in kidney sections from sham and IRI mice treated with ONO-AE3-208 or Perhexiline (maleate). The scale bar corresponds to 50 μm. **(E)** Flow cytometric analysis of the percentage of M1 (CD86^+^) and M2 (CD163^+^) in renal macrophages (Zombie^−^CD45^+^CD11b^+^F4/80^+^cells) in kidney of sham and IRI mice. (*n* = 3 mice per group). *n* = 6 mice per group, Scale bars, 50 μm, Data are means ± s.d.***p < 0.001, **p < 0.01.

Finally, we further confirmed whether the activated EP4 in macrophages exerts renal protection through CPT2-mediated lipophagy *in vivo*. IRI mice were intragastrically administered with Perhexiline (maleate), a CPT2 inhibitor, after IRI treated with vehicle or EP4 agonist. As shown in Figures 7A–D, EP4 agonist significantly ameliorated IRI-induced kidney injury and subsequent renal fibrosis, as evidenced by HE and Masson staining, Western blot and immunohistochemical analyses of the expressions of fibrosis markers (fibronectin and α-SMA). However, the protective effect of EP4 activation was significantly abrogated by the CPT2 inhibitor (Figures 7A–D). Moreover, CPT2 inhibitor markedly suppressed M1 to M2 polarization in the kidney of IRI mice (Figure 7E). These results indicate that EP4 alleviates AKI-to-CKD transition through upregulating CPT2 *in vivo*.

## Discussion

Macrophage-prompted inflammatory and fibrotic responses are key drivers of renal fibrosis and advance the progression of AKI to CKD (Kormann et al., 2020; Lv et al., 2020). However, the molecular mechanisms of macrophages in the AKI–to–CKD transition remain largely unclear, and effective avenues for the clinical treatment of AKI are still lacking. In the present study, we reveal that EP4 was selectively activated in renal macrophages after IRI. Inhibition of EP4 activation aggravated AKI-to-CKD transition, whereas, pharmacologically promoting EP4 activation remarkably ameliorated AKI and its progression to CKD though regulating macrophage polarization. Mechanistically, activation of EP4 prevented AKI-to-CKD transition through inducing CPT2-mediated lipophagy in renal macrophages. Thus, current findings greatly expand our comprehension of the pathogenesis of AKI-to-CKD transition and provide a promising therapeutic target for the treatment against the progression of AKI to CKD.

EP4 is one of the crucial G protein-coupled receptors and its activation regulates the intracellular cyclic cAMP concentration by binding to intracellular G protein and triggers the downstream extracellular signal-regulated kinase 1/2 signals (Patankar et al., 2021). As reported, EP4 plays a vital role in a variety of kidney diseases (Lannoy et al., 2020). Deletion of EP4 significantly aggravated AKI, while promoting EP4 activation could significantly improve AKI (Pan et al., 2022b). Previous studies have demonstrated that pharmacological intervention with EP4 agonist can significantly improve AKI, which are consistent with our studies (Ding et al., 2019). It has been reported that the expression of EP4, not EP2, was significantly increased after renal IRI, which is consistent with our findings, suggesting that EP4 is directly involved in the process of AKI (Pan et al., 2022b). However, several studies reported that EP4 activation enhanced kidney damage (Vukicevic et al., 2006; Mutsaers and Norregaard, 2022). To comprehensively determine the dynamic alterations of EP4 in the process of AKI to CKD, snRNA-seq and flow cytometry were employed in this study to determine the dynamics of EP4 in the kidney tissues from mice with IRI. The snRNA seq analysis and the results of flow cytometry simultaneously demonstrated that EP4 was selectively activated in macrophages, but not in renal tubular epithelial cells, in ischemic kidneys. Further, EP4 expression in CD45^+^CD11b^+^F4/80^+^ macrophages were significantly increased after IRI, which markedly ameliorated AKI and retarded its progression to CKD. However, our study cannot exclude the role of EP4 in renal tubular epithelial cells in the progression of AKI to CKD. We also detected the expression changes of EP4 in renal tubular epithelial cells and macrophages after treatment with agonist or inhibitor, respectively. The results showed that macrophages are more sensitive to the agonist or inhibitor of EP4 (Supplementary Figure S3A). We then detected the downstream target gene of EP4. According to previous studies, both EP4 agonist and PEG2 activate the transcriptional activity of MafB, which is downstream target gene of EP4 (Pan et al., 2022b). Therefore, PCR was applied to detect the expression of MafB in TECs and macrophages separated from the kidney tissues of IRI mice. EP4 inhibitor repress the increase of MafB expression in TECs and macrophages after IRI, whereas EP4 agonist can promote the expression of MafB, which is consistent with the previous results (Supplementary Figure S3B) (Pan et al., 2022b). Interestingly, Ep4 inhibitor led to a slight lipid accumulation and fatty acid metabolism disorder in renal tubular epithelial cells. The results showed that EP4 agonist increased the expression of FAO-related genes, while there are no significant changes between control and EP4 inhibitor-treated HK-2 cells (Supplementary Figure S4A). This indicates that EP4 agonist may also play a protective role by upregulating FAO of TECs, which requires further study. In subsequent work, we would like to further clarify the interplay and underlying mechanism between macrophages and TECs. Nevertheless, Macrophages did pay a pivotal role in the pathogenesis of AKI-CKD transition (Zheng et al., 2021a; Zhang et al., 2021; Meng et al., 2022). Our study found that EP4 activation alleviated macrophage polarization though regulating macrophage polarization, thereby impeded the progression of AKI to CKD, which is also one of the highlights of this study. Taken together, our findings dynamically describe the delicate changes of EP4 expression in macrophages during the progression of AKI to CKD, which may contribute to a better understanding of the role of EP4 during AKI-to-CKD transition.

Accumulated evidence suggested that the observations of metabolic disorders co-existing with renal fibrosis had shed new light on the pathogeny of AKI-to-CKD transition (Miguel et al., 2021). Among these, a dramatic suppression of fatty acid oxidation (FAO) was reported to be essential for the power failure occurring in the tubulointerstitial compartment, leading to renal fibrosis (Kang et al., 2015; Chung et al., 2019). The following studies indicated that enhancing FAO hindered renal fibrosis, since CPT1A-knockin mice manifested declined expression of fibrotic markers, reduced inflammatory response, and diminished macrophage influx in renal fibrosis mouse models (Miguel et al., 2021). Of note, CPT family is the rate-limiting enzyme in FAO signaling pathway. However, the role and mechanism of CPT2 in AKI-to-CKD remain unclear. In the present study, we screened the potential EP4 targets by using bioinformatic predictions and revealed CPT2 as a potent target of EP4 during AKI-to-CKD transition. Subsequent experiments demonstrated that CPT2 mediated EP4-induced lipophagy and the protective effects against the progression of AKI to CKD. Indeed, the expression of Cpt2 is decreased in the results of single cell sequencing, whereas EP4 expression increases in macrophages in response to ischemic kidney injury. This result indicates that the expression of Cpt2 can be regulated by multiple factors. Although our findings demonstrated that CPT2 expression was upregulated by an EP4 agonist and downregulated by an EP4 antagonist, and that the protective effect of EP4 agonist can be abrogated by a CPT2 inhibitor, we cannot deny that CPT2 could also be regulated by other genes. The present study only confirmed the regulatory effect of EP4 on Cpt2 expression in macrophages, which is the limitation of our study. These findings, together with previous reports, collectively reveal that targeting CPT2-mediated FAO may provide a novel therapeutic strategy against AKI-to-CKD transition.

Lipophagy, a selective form of autophagy targeting LDs, selectively recognizes and degrades LDs, which is involved in adjusting cellular lipid metabolism and preserving intracellular lipid homeostasis. Emerging evidence showed that lipophagy in macrophage was closely related to a variety of diseases (Jeong et al., 2018; Robichaud et al., 2021), but the regulatory mechanisms in the progression of AKI to CKD remain poorly understood. Recent studies showed that inhibition of lipophagy was implicated in the pathogenesis of AKI, contributing greatly to renal inflammation and fibrosis (Xiong et al., 2021; Minami and Nakamura, 2022). In this study, we observed that in an IRI-induced AKI-to-CKD transition mouse model, lipophagy was induced by EP4 activation through inducing CPT2 expression in renal macrophages, contributing to the protective effect against AKI-to-CKD progression. Inhibition of lipophagy by 3-MA or CPT2 expression by Perhexiline respectively, could reverse the EP4 activation regulated macrophage polarization. Thus, our study strongly indicates that EP4-CPT2-mediated lipophagy may serve as a potential therapeutic target for the transition of AKI to CKD.

In summary, the present study demonstrates that EP4-CPT2-mediated lipophagy in macrophages plays a protective role during the process of AKI-to-CKD transition. Promoting EP4 activation or restoring the expression of CPT2 in macrophages prevent AKI-to-CKD transition *via* inducing lipophagy and modulating macrophage polarization. These findings not only provide new insights into the molecular mechanisms of AKI-to-CKD transition, but also prompt a potential therapeutic target in progressive AKI.

## Data Availability

The datasets presented in this study can be found in online repositories. The names of the repository/repositories and accession number(s) can be found in the article/Supplementary Material.
