# Supplementary figures and images for: Activation of EP4 alleviates AKI-to-CKD transition through inducing CPT2-mediated lipophagy in renal macrophages
Source: Front Pharmacol. 2022 Nov 16;13:1030800. doi: 10.3389/fphar.2022.1030800 (PMC9709464; doi:10.3389/fphar.2022.1030800)

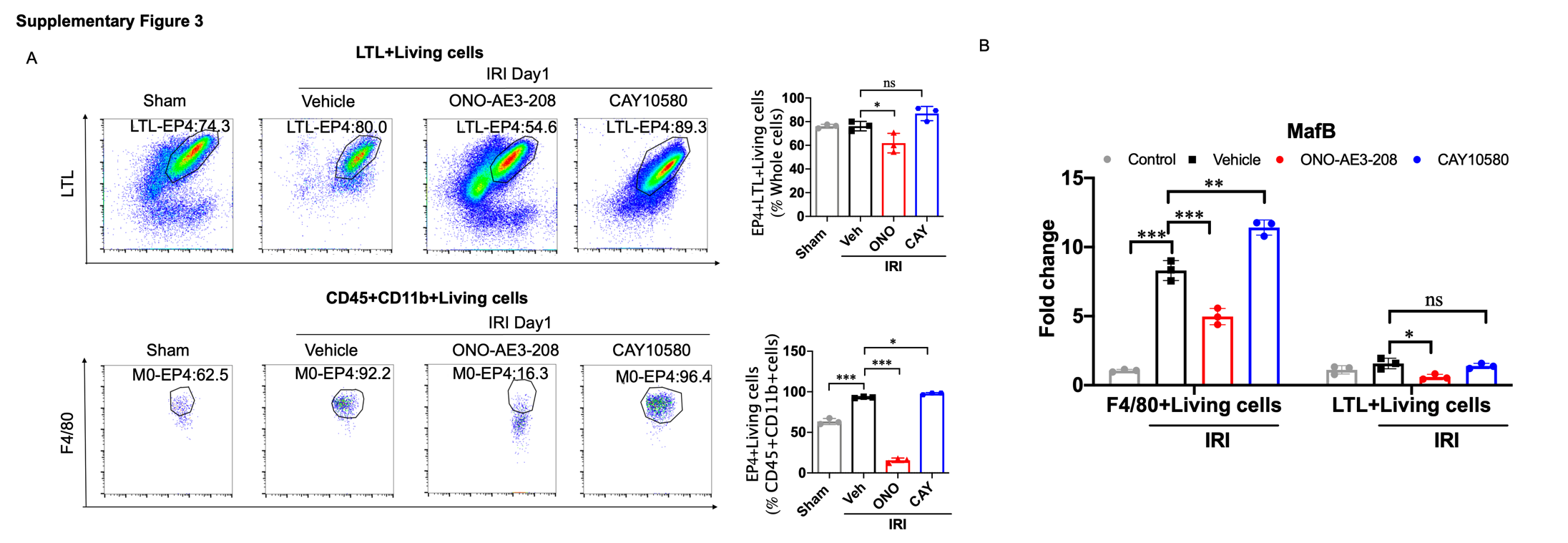

Supplement: Supplementary file 1 [file Image3.TIFF]

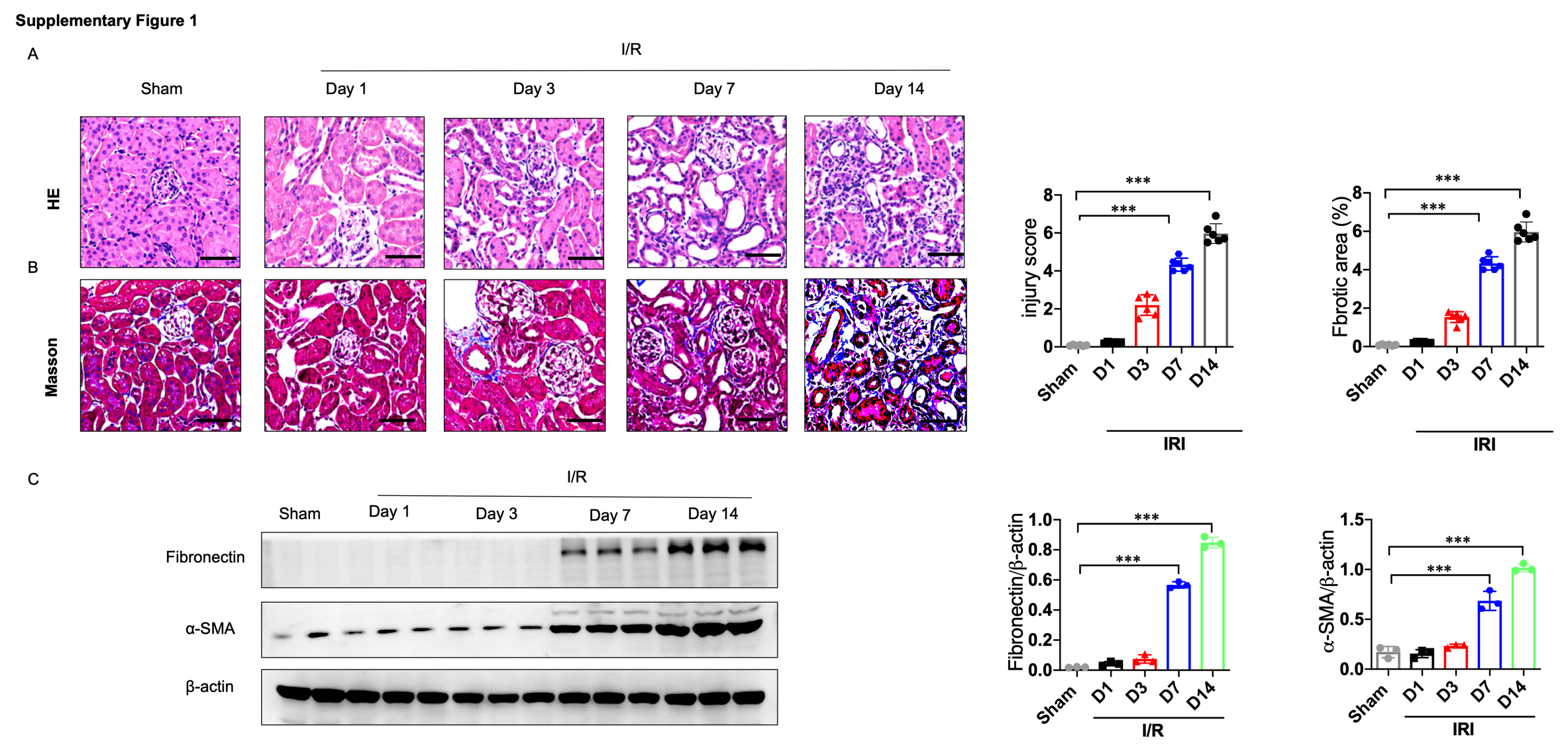

Supplement: Supplementary file 2 [file Image1.TIFF]

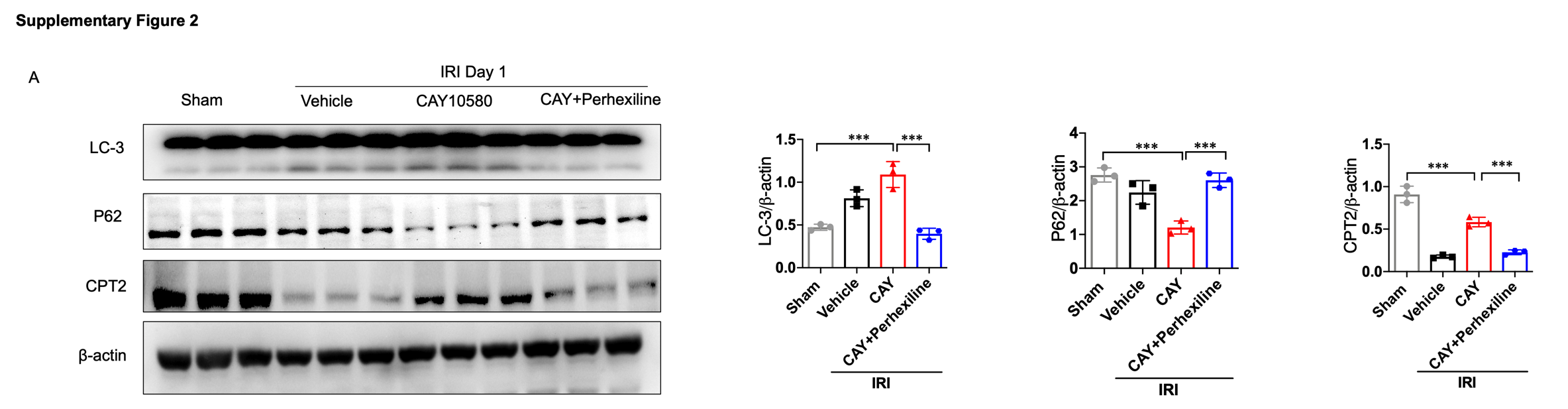

Supplement: Supplementary file 6 [file Image2.TIFF]

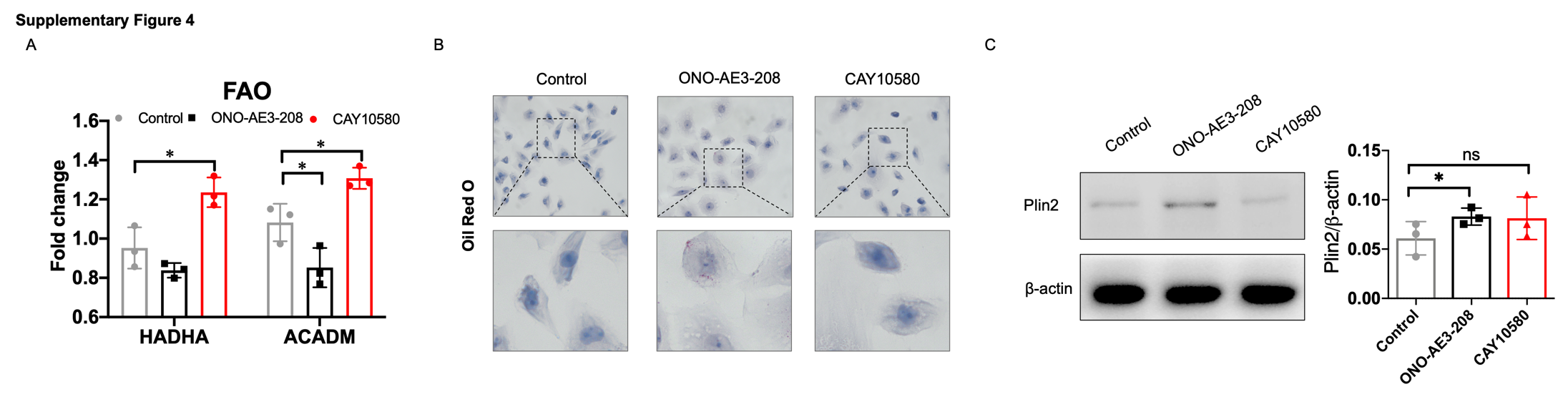

Supplement: Supplementary file 7 [file Image4.TIFF]
