# Supplementary material for: Activation of EP4 alleviates AKI-to-CKD transition through inducing CPT2-mediated lipophagy in renal macrophages
Source: Front Pharmacol. 2022 Nov 16;13:1030800. doi: 10.3389/fphar.2022.1030800 (PMC9709464; doi:10.3389/fphar.2022.1030800)
Supplement: Supplementary file 3 [file Table1.DOC]

**Table S1. Antibody used in the study.**

| Origin | Cat. No | Antibody |
| --- | --- | --- |
| Biolegend | 101263 | CD11b |
| Biolegend | 103131 | CD45 |
| Biolegend | 123114 | F4/80 |
| Biolegend | 105031 | CD86 |
| Biolegend | 423105 | Zombin NIR Fixable viability kit |
| Biolegend | 155306 | CD163 |
| Biolegend | 101320 | CD16/32 |
| Abcam | ab92755 | EP2 |
| Santa | sc-55596 | EP4 |
| CST | 4410S | AF647 |
| Vectorlabs | AL-1323 | LTL |
| Proteintech | 26555-1-AP | CPT2 |
| CST | 26836 | fibronectin |
| CST | AA128 | α-SMA |
| CST | 12741 | LC-3 |
| CST | 5114 | P62 |
| Proteintech | 15294-1-AP | PLIN2 |

**Table S2. The primer sequences used in the study.**

| **Gene** | **Forward** | **Reverse** |
| --- | --- | --- |
| mKim-1 | ACATATCGTGGAATCACAACGAC | ACTGCTCTTCTGATAGGTGACA |
| mNgal | GCAGGTGGTACGTTGTGGG | CTCTTGTAGCTCATAGATGGTGC |
| mCPT2 | CAGCACAGCATCGTACCCA | TCCCAATGCCGTTCTCAAAAT |
| hCPT2 | CATACAAGCTACATTTCGGGACC | AGCCCGGAGTGTCTTCAGAA |
| hHMGCR | TGATTGACCTTTCCAGAGCAAG | CTAAAATTGCCATTCCACGAGC |
| hIL-12 | CCAGAAGGCCAGACAAAC | CCAGGCAACTCCCATTAG |
| hIL-23 | CGTCTCCTTCTCCGCTT | ATCTGAGTGCCATCCTTGA |
| hIL-10 | ACCAAGACCCAGACATCAA | CATTCTTCACCTGCTCCAC |
| hArg-1 | GGAAGTGAACCCATCCCT | GATTACCCTCCCGAGCA |
| hHADHA | ACCGAGGACAGCAACAAGTG | TCAAGCTGCCCAGTCAAGTT |
| hACADM | GGCCGTGACCCGTGTATTAT | CTGCAGCATCGCCCGAA |
| mMafB | CAGGGCTGGTTTGGAATCCT | TTGGCTCAATGGGAGCTCAG |
