# Supplementary material for: Activation of EP4 alleviates AKI-to-CKD transition through inducing CPT2-mediated lipophagy in renal macrophages
Source: Front Pharmacol. 2022 Nov 16;13:1030800. doi: 10.3389/fphar.2022.1030800 (PMC9709464; doi:10.3389/fphar.2022.1030800)
Supplement: Supplementary file 4 [file DataSheet1.PDF]

SUPPLEMENTARY FIGURE LEGENDS

SUPPLEMENTARY FIGURE 1. Establishment of AKI-to-CKD model. (A-B) Representative micrographs of HE and Masson staining of kidney sections from sham and IRI mice at 1, 3, 7, 14 day after ischemia-reperfusion injury. (C) Western blotting analysis for fibronectin and α-SMA protein levels from the kidney of sham and IRI mice at 1, 3, 7, 14 day after ischemia-reperfusion injury. Scale bars, 50 μm. n = 6 mice per group. Data are means ± s.d. ***$p< 0.001$.

SUPPLEMENTARY FIGURE 2. CPT2 inhibition abrogates the protective effect of EP4 activation on AKI-to-CKD. (A) Western blotting analysis for LC-3, P62 and CPT2 protein levels from the kidney of sham and IRI mice treated with CAY10580 or 3-MA. Scale bars, 50 μm. n = 6 mice per group. Data are means ± s.d. ***$p< 0.001$.

SUPPLEMENTARY FIGURE 3. macrophages are more susceptible to EP4 agonist or inhibitor. (A) Flow cytometric analysis of the percentage of EP4 in renal tubular epithelial cells (Zombie$^-$LTL$^+$cells) and macrophages (Zombie$^-$CD45$^+$CD11b$^+$F4/80$^+$cells) in kidney of AKI mice. (B) qRT-PCR analysis of Mafb expression in macrophages and tubular epithelial cells separated from the kidney of sham and IRI mice by Flow sorting. n =3 mice per group. Data are means ± s.d. ***$p< 0.001$, **$p< 0.01$, *$p< 0.05$.

SUPPLEMENTARY FIGURE 4. The effect of EP4 agonist or inhibitor on lipid accumulation and fatty acid metabolism disorder in renal tubular epithelial cells. (A) qRT-PCR analysis of FAO-related genes expression in cultured HK-2 cells treated with ONO-AE3-208 or CAY10580 for 24 h. (B) Representative micrographs of Oil red O (ORO) staining in HK-2 cells treated with ONO-AE3-208 or CAY10580 for 24 h. (C) Western blotting analysis for PLIN2 protein levels f in HK-2 cells treated with ONO-AE3-208 or CAY10580 for 24 h. Scale bars, 50 μm. Data are means ± s.d. *$p< 0.05$.
