# Supplementary material for: Activation of EP4 alleviates AKI-to-CKD transition through inducing CPT2-mediated lipophagy in renal macrophages
Source: Front Pharmacol. 2022 Nov 16;13:1030800. doi: 10.3389/fphar.2022.1030800 (PMC9709464; doi:10.3389/fphar.2022.1030800)
Supplement: Supplementary file 5 [file DataSheet2.docx]

**RAW DATA**

**Raw microscopic images**

<https://www.jianguoyun.com/p/DaPJ5yoQyLX4ChidqNgEIAA>

**Prediction**

<https://www.jianguoyun.com/p/DVfRUZoQyLX4ChieqNgEIAA>

**Fcs**

<https://www.jianguoyun.com/p/DRiJj00QyLX4ChifqNgEIAA>

**Wb**

https://www.jianguoyun.com/p/DWnLhtAQyLX4ChigqNgEIAA

**Prism**

<https://www.jianguoyun.com/p/DW2IjPgQyLX4ChiZqNgEIAA>
